# Effect of a High Intake of Conjugated Linoleic Acid on Lipoprotein Levels in Healthy Human Subjects

**DOI:** 10.1371/journal.pone.0009000

**Published:** 2010-02-03

**Authors:** Anne J. Wanders, Ingeborg A. Brouwer, Els Siebelink, Martijn B. Katan

**Affiliations:** 1 Department of Health Sciences and the EMGO Institute for Health Care Research, VU University Amsterdam, Amsterdam, The Netherlands; 2 Division of Human Nutrition, Wageningen University, Wageningen, The Netherlands; AgroParisTech, France

## Abstract

**Background:**

Trans fatty acids are produced either by industrial hydrogenation or by biohydrogenation in the rumens of cows and sheep. Industrial trans fatty acids lower high-density lipoprotein (HDL) cholesterol, raise low-density lipoprotein (LDL) cholesterol, and increase the risk of coronary heart disease. The effects of trans fatty acids from ruminants are less clear. We investigated the effect on blood lipids of *cis*-9, *trans*-11 conjugated linoleic acid (CLA), a trans fatty acid largely restricted to ruminant fats.

**Methodology/Principal Findings:**

Sixty-one healthy women and men were sequentially fed each of three diets for three weeks, in random order, for a total of nine weeks. Diets were identical except for 7% of energy (approximately 20 g/day), which was provided either by oleic acid, by industrial trans fatty acids, or by a mixture of 80% *cis*-9, *trans*-11 and 20% *trans*-10, *cis*-12 CLA. After the oleic acid diet, mean (± SD) serum LDL cholesterol was 2.68±0.62 mmol/L compared to 3.00±0.66 mmol/L after industrial trans fatty acids (p<0.001), and 2.92±0.70 mmol/L after CLA (p<0.001). Compared to oleic acid, HDL-cholesterol was 0.05±0.12 mmol/L lower after industrial trans fatty acids (p = 0.001) and 0.06±0.10 mmol/L lower after CLA (p<0.001). The total-to–HDL cholesterol ratio was 11.6% higher after industrial trans fatty acids (p<0.001) and 10.0% higher after CLA (p<0.001) relative to the oleic acid diet.

**Conclusions/Significance:**

High intakes of an 80∶20 mixture of *cis*-9, *trans*-11 and *trans*-10, *cis*-12 CLA raise the total to HDL cholesterol ratio in healthy volunteers. The effect of CLA may be somewhat less than that of industrial trans fatty acids.

**Trial Registration:**

ClinicalTrials.gov NCT00529828

## Introduction

Trans fatty acids arise from industrial hydrogenation, and from biohydrogenation in ruminant animals. Industrial trans fatty acids are produced by partial hydrogenation of vegetable and fish oils. Such industrial trans fatty acids raise the total to HDL cholesterol ratio in blood and the risk of coronary heart disease [1]–[5].

Industrial and ruminant fats contain mostly the same species of trans fatty acids, but in different proportions. The major isomers in industrial trans are *trans*-9 18∶1 (elaidic acid) and *trans*-10 18∶1, plus smaller amounts of *trans*-8 18∶1 and *trans*-11 18∶1. In milk and meat the predominant trans fatty acid is *trans*-11 18∶1 (vaccenic acid). In addition, ruminant fats contain small amounts of *cis*-9, *trans*-11 18∶2 (conjugated linoleic acid, abbreviated to CLA in this paper unless otherwise mentioned). CLA is also formed from ingested vaccenic acid in animals and in humans [6]. The proportion of CLA in industrially hydrogenated fats is negligible, but mixtures of *cis*-9, *trans*-11 and *trans*-10, *cis*-12 CLA are produced industrially and sold as supplements.

In rodents, such mixtures of c*is*-9, *trans*-11 and *trans*-10, *cis*-12 CLA isomers improved blood lipid profiles, decreased body fatness, improved insulin sensitivity, and reduced cancer and atherosclerosis risk [7]. When the isomers were studied separately, *cis*-9, *trans*-11 CLA improved blood lipid profile both in hamsters and mice, whereas *trans*-10, *cis*-12 CLA did not affect the blood lipid profile or worsened it [8], [9]. Many of the effects of CLA found in rodents have not been reproduced in other species, including humans. Human studies of the effects of CLA mixtures and of separate c*is*-9, *trans*-11 and *trans*-10, *cis*-12 CLA isomers on lipid profile, weight loss, and insulin resistance have been inconclusive [10], [11]. Most of these studies were uncontrolled trials using CLA supplements. Two controlled dietary intervention studies with diets naturally enriched with c*is*-9, *trans*-11 CLA also did not provide convincing findings, although the ratio of LDL to HDL cholesterol tended to go up on CLA [12], [13].

Nevertheless, CLA is sold widely as a supplement, and levels of c*is*-9, *trans*-11 CLA in ruminant fats have been increased on purpose by altering the animals' feed [14]. We now studied the effects of high doses of c*is*-9, *trans*-11 CLA on lipoprotein levels in a randomized trial in humans.

## Methods

### Ethics Statement

The Medical Ethics Committee of Wageningen University approved the aim and design of the study, which was registered in the NIH clinical trial database (ClinicalTrials.gov number. NCT00529828). The protocol for this trial and supporting CONSORT checklist are available as supporting information; see Checklist S1 and Protocol S1.

### Subjects

We recruited subjects aged 18 to 65 through advertisements in the Wageningen area. Suitable candidates provided written informed consent before screening. We excluded subjects with glycosuria and proteinuria and with concentrations of total cholesterol higher than 6.5 mmol/L or triglycerides higher than 2.3 mmol/L in serum. Subjects were also excluded if they suffered from diabetes or cardiovascular disease, if they used cholesterol lowering or anti-hypertensive medication, had unusual dietary habits including high alcohol intakes, had a body mass index (BMI) >30 kg/m^2^, or were pregnant or lactating.

### Study Design

Our objective was to investigate the effects of c*is*-9, *trans*-11 CLA on serum lipid and lipoprotein concentrations relative to the cis-monounsaturated fatty acid oleic acid (*cis*-9 18∶1). A diet rich in industrial trans fatty acids was provided as a positive control as these are known to raise LDL-cholesterol in comparison with oleic acid. The study was a controlled single blind randomized multiple crossover trial with three consecutive periods of 21 days. The trial ran from September 25 to November 27, 2007. The trial was designed to detect a significant (p<0.05) effect of CLA versus oleic acid on LDL-cholesterol with a power of 80% if the real population effect exceeded 0.13 mmol/L, which is half the effect expected for industrial trans fatty acids [15], [16]. Subjects were randomly divided over all 6 possible diet sequences by an independent researcher using computer-generated numbers.

The study was executed according to methods previously described [17]. We estimated the habitual energy intake of the subjects with a food frequency questionnaire [18]. Diets were designed for 14 levels of energy intake, from 7 to 20 MJ/day, and subjects were allocated to an intake level close to their habitual energy intake. Macronutrients, cholesterol and fiber intakes were based on Dutch nutrition guidelines. On weekdays, subjects consumed a hot meal at lunchtime under our supervision at the metabolic kitchen and dining room of Wageningen University. All other food was provided daily to take away. The supplied foods provided 90% of each subjects' energy requirement. For the remaining 10%, subjects were free to select favorite foods low in fat from a restricted list, which they recorded in a diary. Subjects recorded illnesses, use of medication and any deviations from the diet. We weighed subjects twice a week and adjusted energy intake if bodyweight changed by more than 1 kg. Subjects were asked to maintain their usual pattern of physical activity, and they were not allowed to drink fortified fruit juices, types of coffee that raise cholesterol [19] or to eat more than 10 pieces of liquorice candy a day.

### Study Diets

The diets were intended to be identical except for 7% of total energy as CLA, industrial trans fatty acid or oleic acid (**Table 1**). CLA was provided by a CLA-rich oil (donated by Lipid Nutrition, Wormerveer, The Netherlands) The oil consisted of triglycerides; 78% of the trans fatty acids in it was the *cis*-9, *trans*-11 CLA isomer found in ruminant fat, 17% the *trans*-10, *cis*-12 CLA isomer and 5% other trans fatty acids. Before the study started we performed a safety study in 19 other volunteers. They received 28.9 g CLA-rich oil per day for 3 weeks under strict medical supervision. We found that the treatment had no adverse effects on liver and kidney function tests or other outcomes [20].

**Table 1:** Fatty acid composition of the oils and fats used to produce the experimental margarines and yoghurts.^1^

	High-oleic sunflower oil	Partly hydrogenated vegetable fat	CLA-rich oil
		g/100 g fatty acids	
Saturated fatty acids	8.3±0.1	43.4±0.6	7.7±0.9
Total cis fatty acids	90.0±0.9	15.5±0.2	15.8±0.2
*- Cis-9 C18:1(oleic acid)*	81.9±0.9	11.1±0.2	13.4±0.1
*- Cis-9,cis-12 C18:2 (linoleic acid)*	7.6±0.0	0.3±0.0	2.2±0.1
Total trans fatty acids (other than CLA)	0.3±0.4	38.9±0.1	0.8±0.9
Total CLA	0.0±0.0	0.2±0.1	73.7±0.6
*- Cis-9,trans-11 CLA*	0.0±0.0	0.0±0.0	56.9±0.4
*- Trans-10,cis-12 CLA*	0.0±0.0	0.0±0.0	12.8±0.0

1 CLA, Conjugated Linoleic Acid.

Fatty acid composition (means ± SD) from analyses of one sample taken before and one sample taken after the study.

NIZO Food Research (Ede, The Netherlands) manufactured margarines and yoghurt drinks enriched with the special oils and fats to our specifications. The fat in the oleic acid margarine consisted of 82% high-oleic sunflower oil (Aldoc BV, Schiedam, The Netherlands) and 18% of a standard hard stock, an interesterified mixture of palm oil and palm kernel fat (Unilever Research & Development, Vlaardingen, The Netherlands). The fat in the CLA margarine contained 25% CLA-rich oil, 10% sunflower oil, 47% high-oleic sunflower oil and 18% standard hard stock, and the fat of the industrial trans margarine consisted of 65% partially hydrogenated vegetable fat (Melano, FUJI Oil Europe, Gent, Belgium), 10% sunflower oil and 25% high-oleic sunflower oil. In addition, yoghurt drinks were produced by enriching fat-free yoghurt with 5 g high oleic sunflower oil, 5 g CLA-rich oil or 5 g partially hydrogenated vegetable fat per 100 ml, respectively. The margarines were used as a spread, and as an ingredient in bread, cookies, sauces, and gravies. We bought other food items commercially.

### Diet Composition

Duplicate diets were collected daily; they were stored at −20°C and analyzed for protein, fat, fatty acids, dry matter, ash, dietary fiber and digestible carbohydrate [21]–[23]. CLA isomers were separated by gas chromatography on a Sil-88 column and other fatty acids on a WAX-58 column (Varian, Middelburg, The Netherlands). The nutrients in the free-choice items were calculated (NEVO, 2006) and added to the analyzed values.

### Blood Measurements

We took blood samples after an overnight fast on days 19 and 21 of each of the three diet periods and stored serum and plasma at −80°C. Samples were taken at the same time on every blood-sampling day to minimize within-subject variation. All six blood samples of one subject were analyzed within the same run to eliminate interassay variation. Fatty acids in plasma cholesteryl esters were analyzed in the samples taken on day 19 of each period as described above [24]. The results for CLA isomers and other fatty acids were combined and expressed as a proportion by weight of all fatty acids detected. Apolipoprotein B was measured on a Behring Nephelometer Analyzer II (Dade Behring, Marburg, Germany) in EDTA plasma pooled per subject per period. Total and HDL-cholesterol and triglycerides were determined enzymatically on a Synchron LX20 (Beckman Coulter, Mijdrecht, The Netherlands), LDL-cholesterol was calculated [25].

### Statistical Analysis

We defined the outcome variables and the statistical analysis scripts in detail before the results of the measurements were known. Treatment codes were broken after data-analysis was completed. For each subject, blood lipid outcomes were averaged for the two blood samples per diet. All data are reported as mean ± standard deviation (SD), 95% confidence interval, or % change compared to reference group. Data were analyzed by the mixed linear model procedure in SPSS version 15.0 (SPPS, Chicago, Ill) with serum lipids as dependent and diet and period as independent variables. Carryover effects were tested by introducing a treatment-by-period interaction term, and heterogeneous compound symmetry was selected as covariance structure [26]. When the analyses indicated a significant effect of diet (p<0.05), Least Significant Difference t-test was used for pair wise comparisons of the CLA and industrial trans fatty acids diets versus the control oleic acid diet. After the codes had been broken we became aware of the gender effect reported by Chardigny et al. [27]. We therefore tested gender differences by including sex and a sex-by-treatment interaction term in the model.

## Results

**Figure 1:**
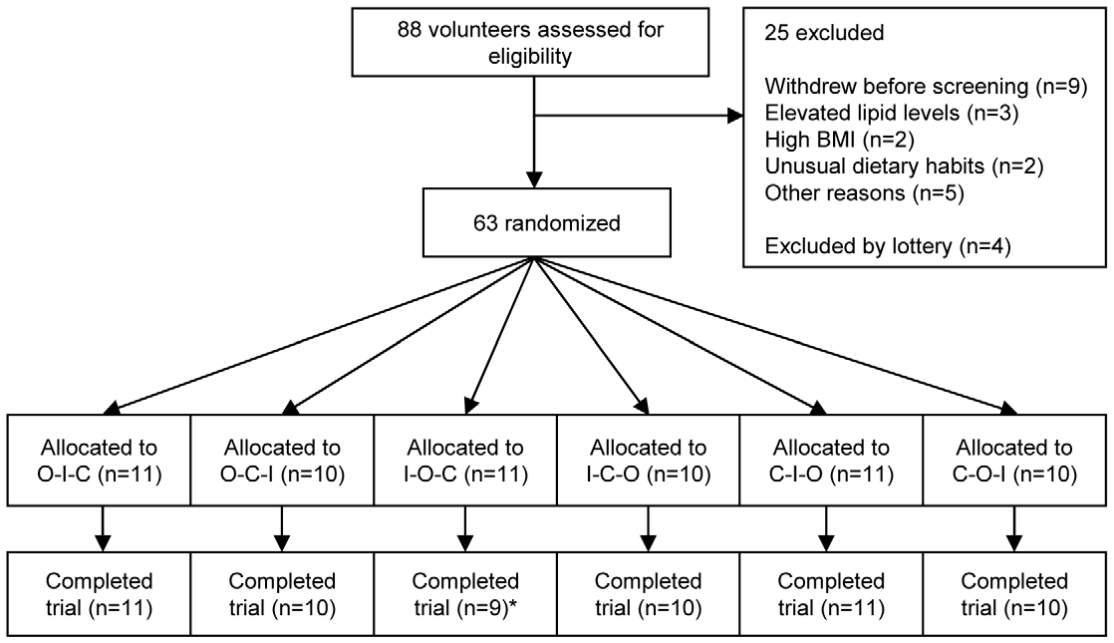
Flow chart of a randomized cross-over trial of the effect of Conjugated Linoleic Acid (CLA) on lipoprotein levels in 61 healthy subjects. Each intervention period lasted 3 weeks; O, Oleic acid diet; I, Industrial trans fat diet; C, CLA diet. * 2 subjects dropped out early in the study, one for personal reasons and one because of illness, both unrelated to the trial. These subjects were not included in the analysis.

Eighty-eight apparently healthy volunteers applied for enrolment into the study (**Figure 1**). Nine withdrew before the pre-study screening visit and 12 proved ineligible. Major reasons for exclusion were elevated lipid levels (n = 3), high BMI (n = 2) and unusual dietary habits (n = 2). Out of the 67 eligible volunteers, we excluded four by lottery. Sixty-three apparently healthy subjects started the study. One man withdrew at 6 days for personal reasons and one woman at 20 days due to illness not related to the study. **Table 2** gives the baseline characteristics of the 61 participants who completed the study.

**Table 2:** Baseline characteristics at the pre-study screening of all subjects who completed the study (n = 61).

Characteristics	
Age (yr)	30.9±13.7^1^
Gender: male (n)	25 (41%)^2^
Height (m)	1.75±0.09
Weight (kg)	70.2±12.5
Body Mass Index (kg/m^2^)	22.8±3.2
Energy intake (MJ/d)	10.3±3.3
Total cholesterol (mmol/L)	4.54±0.77
HDL-cholesterol (mmol/L)	1.31±0.36
LDL-cholesterol (mmol/L)	2.87±0.68
Total to HDL cholesterol ratio	3.66±0.96
LDL to HDL cholesterol ratio	2.35±0.86
Serum triglycerides (mmol/L)	0.98±0.45

1 Values are means ± SD.

2 Values are numbers (n); percentage in parentheses.

### Diets and Dietary Adherence

The intake of total protein, fat, carbohydrates, alcohol, cholesterol and dietary fiber did not differ between diets (**Table 3**). At an energy intake of 11 MJ (2627 kcal) per day, the CLA diet provided 26.8 g of CLA isomers, while the industrial trans diet supplied 21.8 g of *trans* C18:1 per day.

**Table 3:** Mean daily intakes of energy and nutrients according to chemical analysis of duplicates of the diets.^1^

	Oleic acid diet	Industrial trans diet	CLA diet
**Energy intake (MJ/day)**	10.6	10.8	10.7
**Kcal/day**	2532	2568	2553
**Fat (% of energy)**	39.7	40.1	39.7
Saturated fatty acids	10.5	13.8	11.3
*- C12:0 (lauric)*	0.7	0.2	0.8
*- C14:0 (myristic)*	0.9	0.7	1.1
*- C16:0 (palmitic)*	5.7	9.7	6.3
*- C18:0 (stearic)*	2.0	2.3	1.9
Total cis fatty acids	27.4	17.1	17.7
*- Cis-9 C18:1 (oleic)*	23.1	11.3	13.4
*- Cis-9,cis-12 C18:2 (linoleic)*	3.2	4.1	3.4
Total trans fatty acids	0.2	7.5	9.1
*- Total trans C18:1*	<0.1	7.3	<0.1
*- Trans-9 C18:1(elaidic)*	<0.1	3.1	<0.1
*- Trans-10 C18:1*	<0.1	1.5	<0.1
*- Trans-11 C18:1(vaccenic)*	<0.1	0.8	<0.1
*- Total CLA*	0.1	0.1	9.0
*- Cis-9,trans-11 CLA*	0.1	0.1	6.9
*- Trans-10,cis-12 CLA*	0.0	0.0	1.5
**Protein (% of energy)**	12.6	11.8	12.8
**Carbohydrates (% of energy)**	46.3	46.7	46.0
**Alcohol (% of energy)**	1.4	1.4	1.5
**Dietary fiber (g/MJ)**	3.0	2.9	2.9
**Cholesterol (mg/MJ)**	22.3	22.8	21.9

1 CLA, Conjugated Linoleic Acid.

The nutrients of the free-choice low fat items (less than 10 energy percent) were calculated (NEVO, 2006) and added to the analyzed values.

The fatty-acid composition of plasma cholesteryl esters confirmed the subjects' adherence to the diets. At the end of the oleic acid diet period, *cis* C18:1 fatty acids comprised 24.7±4.3 g/100 g fatty acids. On the CLA and industrial trans diet this was 17.6±3.4 and 18.5±3.8 g/100 g, respectively. The sum of CLA isomers in plasma cholesteryl esters was 4.8±1.4 g/100 g after the CLA diet, 1.4±0.7 g/100 g after the oleic acid diet and 1.5±0.6 g/100 g after the industrial trans diet.

Diaries kept by the subjects revealed only minor deviations from the protocol that were unlikely to affect outcomes. Six subjects reported nausea for 1 or 2 days on the CLA and industrial trans fatty acid diets, and 2 while on the oleic acid diet. Three reported diarrhea for 1–2 days while on the CLA diet.

Over the 63 days of the trial, average body weight fell by 0.6±1.7 kg. Body weight at the end of the oleic acid diet differed 0.0±0.9 kg from that after the CLA diet and −0.1±0.9 kg from that after the industrial trans fatty acid diet. Caloric intakes did not differ between the 3 diets (p = 0.998).

### Serum Lipids and Lipoproteins

The total trans fatty acid intake on the CLA diet was 1.6% of energy higher than on the industrial trans fatty acid diet while the saturated fatty acids intake was 2.5% of energy lower (Table 3).

In order to provide an unbiased comparison between industrial trans fatty acids and CLA, we corrected the serum lipid responses for these differences in intake using the equations of Mensink et al. [16]. The corrected levels were only slightly different from the original levels (**Table 4**), and the statistical significance of the changes relative to oleic acid was the same whether we used corrected or uncorrected levels.

**Table 4:** Mean (± SD) values for serum lipid, lipoprotein cholesterol and apoB levels at the end of the three dietary periods (n = 61), and means (95% Confidence Intervals) for individual differences between diets.^1^

	Oleic acid diet	Industrial trans diet uncorrected	Industrial trans diet 2	CLA diet	Industrial trans 2 minus oleic acid	CLA minus oleic acid	CLA minus Industrial trans 2
	Mean ± SD	Mean ± SD	Mean ± SD	Mean ± SD	Change 3 (95% CI)	Change 3 (95% CI)	Change 3 (95% CI)
				mmol/L			
Total cholesterol	4.42±0.70	4.77±0.75	4.72±0.75	4.62±0.82	0.31 4	0.20 4	−0.10 6
					(0.22 to 0.39)	(0.11 to 0.30)	(−0.20 to −0.01)
HDL-cholesterol	1.31±0.29	1.28±0.29	1.26±0.29	1.25±0.30	−0.05 4	−0.06 4	0.00
					(−0.08 to −0.03)	(−0.09 to −0.03)	(−0.03 to 0.03)
LDL-cholesterol	2.68±0.62	3.02±0.66	3.00±0.66	2.92±0.70	0.31 4	0.23 4	−0.08 6
					(0.24 to 0.38)	(0.16 to 0.31)	(−0.15 to 0.00)
Total to HDL ratio	3.48±0.75	3.88±0.89	3.89±0.89	3.83±0.92	0.40 4	0.35 4	−0.06
					(0.32 to 0.49)	(0.25 to 0.44)	(−0.15 to 0.04)
LDL to HDL ratio	2.14±0.68	2.48±0.78	2.50±0.78	2.44±0.78	0.35 4	0.30 4	−0.05
					(0.28 to 0.43)	(0.22 to 0.38)	(−0.13 to 0.03)
Triglycerides	0.93±0.33	1.05±0.41	1.08±0.41	0.99±0.42	0.15 4	0.05	−0.10 5
					(0.09 to 0.21)	(−0.01 to 0.12)	(−0.16 to −0.03)
				mg/dl			
Apo-B	70.8±14.6	78.2±15.7	76.0±15.7	74.1±16.5	5.2 4	3.3 4	−1.9 6
					(3.5 to 6.9)	(1.6 to 5.1)	(−3.6 to −0.1)
ApoB to LDL ratio	266±28	261±27	256±27	256±27	−10.4 4	−9.9 4	0.5
					(−15.3 to −5.6)	(−14.6 to −5.2)	(−4.1 to 5.2)

1 The subjects consumed each diet for 3 weeks each, in random order.

2 Levels on industrial trans fatty acids were recalculated to correct for the slightly higher intake of saturated and the slightly lower intake of total trans and cis fatty acids on the industrial trans diet than on the CLA diet. Corrections were made using the equations of Mensink et al. [16].

3 Variables were analyzed with a mixed linear model, with diet as the repeated factor within subjects and period as a fixed effect. P<0.05 was considered to be a significant effect. When significant, Least Significant Differences t-test procedure was used for pair wise comparisons:

4 p<0.001.

5 p<0.015.

6 p<0.05.

The LDL-cholesterol level was 11.6% (p<0.001) higher after the industrial trans diet and 8.7% (p<0.001) higher after the CLA diet than after the oleic acid diet (Table 4). LDL-cholesterol levels were higher in 53 subjects after the CLA diet and in 55 subjects after the industrial trans fatty acid diet. The ratio of total to HDL cholesterol was 11.6% higher after the industrial trans fatty acid diet (p<0.001), and 10.0% higher after the CLA diet (p<0.001). The apolipoprotein B level was higher on both the industrial trans (7.4%; p<0.001) and the CLA diet (4.7%; p<0.001). The level of triglycerides was significantly higher after the industrial trans fatty acid diet (16.1%; p<0.001) but not after the CLA diet (5.7%; p = 0.085). The responses of blood lipids to industrial trans fatty acids were close to those predicted by the Mensink equations [16]. For instance, we found an increase in LDL-cholesterol of 0.33 mmol/L uncorrected, or 0.31 mmol/L after correction, while the equations predicted an increase of 0.35 mmol/L. HDL-cholesterol decreased by 0.03 mmol/L uncorrected, or 0.05 mmol/L after correction, while the predicted decrease was 0.06 mmol/L.

We also compared the two types of trans fatty acids with each other. After the industrial trans fatty acid diet the LDL-cholesterol level was 2.6% higher than after CLA (p = 0.049). Levels of serum triglycerides (8.9%; p = 0.004) and apolipoprotein B (2.5%; p = 0.041) were also higher after the industrial trans than after the CLA diet. The effects of industrial trans and CLA on the total to HDL cholesterol and the LDL to HDL cholesterol ratio did not differ significantly. There were no important carry over or period effects.

### Gender

In our study, men were more sensitive to the effects of trans fatty acids than women. Sex-by-treatment interaction was found for the total to HDL cholesterol ratio (p = 0.001), LDL to HDL cholesterol ratio (p = 0.007), and triglyceride levels (p = 0.014). After the CLA diet the ratio of total to HDL cholesterol was 0.55±0.46 higher in men and 0.20±0.27 higher in women (p = 0.002 for difference) than on the oleic acid diet. The ratio of LDL to HDL cholesterol was 0.45±0.36 higher in men and 0.20±0.23 higher in women (p = 0.003). On the industrial trans fatty acid diet ratios also increased more in men than in women. After the CLA diet triglyceride levels in men were 0.16±0.34 higher and in women 0.02±0.17 lower than on oleic acid.

## Discussion

We found that a high intake of *cis*-9, *trans*-11 CLA plus a smaller amount of *trans*-10, *cis*-12 CLA raised LDL-cholesterol and lowered HDL-cholesterol levels in healthy humans. The effect of CLA was somewhat less than that of industrial trans fatty acids.

### Strengths and Limitations

Our study had several strengths. Food intake was strictly controlled, the composition of the diet was verified by chemical analysis, and adherence was evidenced by changes in blood fatty acids. The statistical power was high and the confidence intervals of the outcomes were narrow (Table 4). Lack of power caused by the use of low doses is a likely explanation why previous studies have failed to detect the effect of CLA on HDL and LDL-cholesterol unambiguously [28], [29]. Our use of both a cis-monounsaturated and an industrial trans fatty acid diet facilitate comparison of our outcomes with those of other studies. Finally, we employed both male and female volunteers.

Our study also had limitations. First, we fed doses of trans fatty acids which cannot be reached with a normal diet. We used a high intake of CLA because otherwise the number of subjects needed to define the outcomes with sufficient precision would have become prohibitive. At an intake of 3.6 grams per day (∼1.3% energy), which was typical in previous studies, we would have needed 617 volunteers to obtain a significant effect of CLA on LDL-cholesterol. Second, our treatments lasted only 3 weeks. Effects of other fatty acids on lipid and lipoprotein levels stabilize within 2 weeks after a change in dietary fatty acids [30], [31], but the long-term effects of CLA might still differ from what we found in this trial. Third, the CLA diet did not contain pure *cis*-9, *trans*-11 CLA but also 1.5% of energy (4.4 g/d) as *trans*-10, *cis*-12 CLA. It has been suggested that the latter has a more unfavorable effect on blood lipids than the *cis*-9, *trans*-11 CLA found in ruminant fats [32]. However, this has not been confirmed in other studies [28], [29]. The effect of the ruminant *cis*-9, *trans*-11 CLA isomer may be less than suggested by our data because of the presence of *trans*-10, *cis*-12 CLA, but it is unlikely that the entire effect of the CLA oil was due to the relative small amount of *trans*-10, *cis*-12 CLA. Finally, our subjects were young, lean and healthy. Studies of industrial trans fatty acids yielded similar effects on blood lipids in different populations, but still the effect of CLA on lipid and lipoprotein levels seen here needs verification in the elderly and in patients at risk for cardiovascular disease.

### Comparison with Previous Studies

Previous studies of CLA mostly compared the effects of approximately 3.6 grams of CLA per day with a placebo, with no control of food intake. Effects on lipids and lipoprotein levels were mostly not significant [28], [29], [33]–[41]. However, closer inspection shows that most of these studies observed an increase of the LDL to HDL cholesterol ratio, even though that effect failed to reach statistical significance (Brouwer et al., submitted). Controlled diets high in CLA were provided in two studies. Desroches et al. found that butter high in CLA and other natural trans fatty acids significantly raised plasma cholesterol and the LDL to HDL cholesterol ratio relative to control butter [12]. A similar study from Tricon et al. [13] also found a small but significant rise in the LDL to HDL cholesterol ratio on dairy products high versus low in CLA and other dairy trans fatty acids. In both studies, some 60 to 80% of the animal trans consisted not of CLA but of vaccenic acid (*trans*-11 18∶1), a trans fatty acid also found in partially hydrogenated industrial fats. Our study shows that *cis*-9, *trans*-11 CLA, which is exclusively found in milk and body fat of ruminant animals, accompanied by a smaller dose of *trans*-10, *cis*-12 CLA, also raises the LDL to HDL cholesterol ratio.

In our study, men showed greater responses of some lipids and lipoproteins to both CLA and to industrial trans fatty acids than women. In contrast, the TRANSFACT trial, a study comparing ruminant trans fatty acids with industrial trans fatty acids, found a greater response to ruminant trans fatty acids in women than in men [27]. Other trials found no differences between men and women in the response of blood lipids to industrial trans fatty acids [42]. Both the larger effect in men seen in our study and the smaller effect seen in the TRANSFACT study may therefore be due to chance. We could also assume separate physiologic mechanisms between men and women in response to both CLA and industrial trans fatty acids. These hypothetical mechanisms would cause CLA to have a larger effect and would cause the ruminant trans fatty acids used in TRANSFACT to have a smaller effect on blood lipids in men than in women. However, in view of the sparsity of data we would rather invoke chance fluctuations than a multitude of mechanisms with opposite actions.

### Clinical Relevance

The effect of CLA provided by usual intakes of milk and meat on blood lipids is negligibly small even if it lowers HDL- and raise LDL-cholesterol to the extent predicted by our study. Mozaffarian et al. [5] calculated that increasing trans fatty intake by 1% of energy increases the risk of cardiovascular disease by 3 to 12%. The average dietary intake of 0.3 g of *cis*-9, *trans*-11 CLA a day, or 0.1% of energy, in Europe [43], [44] would then increase the risk of cardiovascular disease by only 0.3 to 1.2%.

Much higher amounts of CLA are taken in the form of supplements. An intake of 3 g/day of a CLA mixture, as recommended by manufacturers of CLA supplements, could theoretically increase the risk of cardiovascular disease by 3 to 12%. CLA supplements should therefore have clear-cut other health benefits to justify their intake. At present, these benefits have not been firmly established. Moreover, recent changes in dairy cattle feeding have led to milk with a lower content of saturated fatty acids and a higher content of *cis*-9, *trans*-11 CLA and other dairy trans fats [45]. Our data suggest that the effect of these changes on heart disease risk in consumers of milk and meat fat are at the very least equivocal.

### Conclusion

Our results indicate that a high intake of *cis*-9, *trans*-11 CLA, a trans fatty acid largely restricted to milk and meat, plus a smaller amount of *trans*-10, *cis*-12 CLA raises LDL- and lowers HDL-cholesterol in humans. The effect of CLA on the LDL to HDL cholesterol ratio and the total to HDL cholesterol ratio is comparable with that of the artificial trans fatty acids that arise during industrial hydrogenation of vegetable oils.

## Supporting Information

Checklist S1 Consort Checklist
(0.06 MB DOC)

Protocol S1 Trial Protocol
(0.42 MB DOC)
