# Supplementary material for: Effect of a High Intake of Conjugated Linoleic Acid on Lipoprotein Levels in Healthy Human Subjects
Source: PLoS One. 2010 Feb 3;5(2):e9000. doi: 10.1371/journal.pone.0009000 (PMC2815780; doi:10.1371/journal.pone.0009000)
Supplement: Protocol S1 — Trial Protocol (0.42 MB DOC) [file pone.0009000.s002.doc]

Health effects of CLA versus industrial trans fatty acids

Protocol for a controlled dietary trial

Version 5

Prof Dr Martijn B Katan

Institute for Health Sciences

De Boelelaan 1085

1081 HV Amsterdam

The Netherlands

Tel: +31 20 598 2610

E-mail: martijn.katan@falw.vu.nl

Dr Ingeborg A Brouwer

Institute for Health Sciences

De Boelelaan 1085

1081 HV Amsterdam

The Netherlands

Tel: +31 20 598 7702

Fax: +31 20 5986940

E-mail: ingeborg.brouwer@falw.vu.nl

or

Div. of Human Nutrition

Wageningen University

Bomenweg 2

6703 HD Wageningen

The Netherlands

Contents

Introduction [3](#__RefHeading___Toc230850808)

Objective of the trial [5](#__RefHeading___Toc230850809)

Pre-study: CLAxon [5](#__RefHeading___Toc230850810)

Design of CLARINeT [6](#__RefHeading___Toc230850811)

Subjects [6](#__RefHeading___Toc230850812)

Hypothesis, statistical power, and number of subjects [6](#__RefHeading___Toc230850813)

Diet composition [7](#__RefHeading___Toc230850814)

Analyses [8](#__RefHeading___Toc230850815)

Design of the cross-over study [9](#__RefHeading___Toc230850816)

Statistical analyses [9](#__RefHeading___Toc230850817)

Investigators [9](#__RefHeading___Toc230850818)

Timelines [10](#__RefHeading___Toc230850819)

Collaboration with Lipid Nutrition [10](#__RefHeading___Toc230850820)

References [11](#__RefHeading___Toc230850821)

Appendix 1: Power table [14](#__RefHeading___Toc230850822)

Appendix 2: available partially hydrogenated industrial fats [16](#__RefHeading___Toc230850823)

Appendix 2: available partially hydrogenated industrial fats [16](#__RefHeading___Toc230850824)

## Introduction

High intakes of trans fatty acids increase the risk of coronary heart disease and authorities worldwide are attempting to limit their intake. However, there are two sources of trans fatty acids with possibly different effects on health. So-called ‘industrial’ trans fatty acids are produced by partial hydrogenation of vegetable and fish oils. The unfavourable effects of these trans fatty acids are well established: they raise LDL-cholesterol, depress HDL cholesterol, and are associated with increased coronary heart disease (fig. 1A) [Ascherio, 1999]. Trans fatty acids also occur naturally in the dairy and body fat of ruminant animals including cows, and these ruminant trans fatty acids are now the dominant form in the European diet. The effects of dairy trans fatty acids on blood lipids in humans have not been studied, and their intake is associated with less rather than more coronary heart disease in several studies (fig. 1B) [Willett WC, 1993; for a review see Weggemans, 2004]

*Figure 1. Intake of trans fatty acids at baseline and risk of coronary heart disease in prospective cohort studies, by source of trans fatty acids. Fig 1A, trans fatty acids from partially hydrogenated oils. Fig. 1B, trans fatty acids from dairy, beef and mutton*

Up to 50% of the trans fatty acids in dairy fat consists of vaccenic acid (C18:1trans11/n-7). CLA resembles vaccenic acid because it also has a trans 11 bond (C18:2 trans 11/cis 9). Furthermore, in humans vaccenic acid is converted into CLA (trans 11, cis 9) by the delta-9 desaturase enzyme [Kuhnt et al. 2006]. Trans fatty acids from partially hardened vegetable oils contain a range of positional isomers, with C18:1trans9/n-9 (elaidic acid) and C18:1trans10/n-8 most prominent isomers. Vaccenic acid is a minor constituent of hardened vegetable oil; CLA is not present.

The lack of information about the health effects of dairy trans has led different authorities to adopt different policies. Thus, Danish law prohibits trans fatty acids in food fats at level >2%, but trans fatty acids from dairy and meat are exempt. In contrast, the European Food Safety Authority did not recognize a distinction between dairy and industrial trans [ www.efsa.eu.int/science/nda/nda_opinions/588/opinion_nda09_ej81_tfa_en1.pdf ].

Dairy fats contain 3-6 % trans fatty acids. Although this is a small proportion, dairy and meat are now the number one source of trans fatty acids in countries where intake of industrial trans fatty acids has gone down [Hulshof, 1999]. It is thus urgent to determine whether the effects of dairy trans and “industrial” trans fatty acids are different. For this purpose we plan to use conjugated linoleic acid (CLA). If CLA would prove to lack the adverse effect of other trans fatty acids that would help us to pinpoint the mechanism through which trans fatty acids raise cardiovascular risk, and it would provide industry with a useful and healthy food ingredient. If on the other hand dairy and industrial trans are similarly harmful this would encourage producers to reduce trans in milk fat. Present intake of ruminant trans is typically 1-2% of energy; if this is reduced by 1% the predicted fall in coronary heart disease mortality in the population would be between 3 and 10% [Mozaffarian, 2006].

CLA has received attention because of its beneficial metabolic and anti-obesity action in animals. In mice, CLA decreased body fat [Park et al. 1997; West et al. 1998], and in male ZDF rats, it improved insulin sensitivity [Houseknecht et al. 1998]. Furthermore, studies suggest that CLA may also have anticarcinogenic effects [Ha et al. 1987] as well as lipid - and atherosclerosis - reducing properties [Lee et al. 1994]. Human studies on effects of CLA on weight loss, insulin resistance and lipid profile show less consistent results [Taylor et al. 2006; Riserus et al. 2001 and 2002; Thom et al. 2001; Gaullier et al. 2004 and 2005; Blankson et al. 2000; Kreider et al. 2002; Kamphuis et al. 2003; Smedman et al. 2001; Belury et al. 2003]. Salas-Salvadó et al. reviewed the literature in 2006 and concluded that further studies are needed to confirm or deny possible effects on weight and body composition and to investigate possible undesirable effects on lipid or glucose metabolism [Salas-Salvadó et al. 2006]. The main reason for the lack of consistency for the studies on lipid effects is lack of power; most studies were too small to show a clear effect and used amounts of CLA of which no significant effects on cholesterol concentrations can be expected.

Here we propose a trial to settle this issue with the use of CLA. The main trans fat in dairy products is vaccenic and this would therefore be our first choice for testing effects of dairy trans. However, not enough pure dairy trans fat is available for clinical trials; a study in hamsters already consumed most of the world’s supply of vaccenic acid at that time [Meijer, 2001]. Therefore, we have chosen another trans fat that is present in dairy products, namely CLA. Nowadays several feeding and farming techniques for ruminant animals are used to increase the content of CLA in butter, margarine, eggs and milk. Furthermore, CLA supplements are freely available on the market.

Lipid Nutrition (Wormerveer) will provide sufficient cis9,trans11 CLA the main form of CLA in dairy. We want to determine whether CLA affects LDL and HDL levels in humans. In order to have enough power to pick up small effects we will feed a high dose and we can then extrapolate back to effects of lower intakes. Effects (or the lack of them) seen with CLA might also be relevant in evaluating the effect of vaccenic acid. As the maximum number of subjects we can include in a controlled dietary trial is 60 we need to give about 7 energy percent of CLA per day to reach sufficient power. It is not possible to test the effect of CLA acid on coronary heart disease itself, but a well-established intermediate risk factor is the effect on LDL and HDL. If CLA raises LDL and lowers HDL then ruminant trans fatty acids probably raise the risk for heart disease. Conversely, if CLA does not affect blood lipoprotein levels this confirms the epidemiological studies that saw no adverse effects of ruminant trans intake.

This trial may also show if trans fatty acids affect inflammation, which could be an important intermediate between trans fatty acids and coronary heart disease. [Baer, 2004]

## Objective of the trial

To investigate what the effect is of CLA on blood lipoproteins, inflammatory markers, blood pressure and insulin status in human volunteers relative to industrial trans fatty acids and to oleic acid

## Pre-study: CLAxon

The knowledge on health effects of CLA was limited before the start of the CLA study. Therefore, a pre-study was performed to investigate the effects of intake of CLA on liver- and kidney function.

The pre-study included 20 healthy subjects aged 18-60 years with normal liver- and kidney function. Subjects took daily approximately 20 grams of CLA, which is a comparable amount as will be used in the main study. Blood was collected at day 0, 3, 7, 10, 16 and 21 of the intervention period and 1 and 3 weeks after the intervention period.

**Results CLAxon**

None of the subjects showed blood values that indicated an unfavorable effect of intake of 20 grams of CLA on liver- or kidney function. One subject stopped the study because of dislike of the study products.

Creatinine clearance was significantly different at day 21 compared with day 0 (table 1). However, this effect was in a beneficial direction and cannot be considered clinically relevant.

Table 1: Paired t-test for effects on liver- and kidney function parameters of 19 subjects baseline versus after 21 days of intake of 20 grams of CLA.

| Parameter | Mean  (SD) day 0 | Mean (SD) day 21 | Delta day 21 – day 0 | 95% Confidence Interval of the Difference |  | P * |
| --- | --- | --- | --- | --- | --- | --- |
|  |  |  |  | Lower | Upper |  |
| Creatinine clearance | 70.1 (5.9) | 68.4 (6.5) | 1,7 | ,03 | 3,31 | ,046 |
| ALAT | 13.9 (5.6) | 14.5 (3.7) | -,53 | -2,68 | 1,63 | ,615 |
| ASAT | 17.9 (3.1) | 19.0 (5.1) | -1,1 | -3,73 | 1,52 | ,388 |
| Alkaline Phosphatase | 58.9 (13.2) | 60.1 (13.1) | -1,2 | -4,30 | 1,85 | ,417 |
| Bilirubine | 11.3 (3.7) | 11.1 (4.7) | ,17 | -1,01 | 1,34 | ,771 |

* Two sided t-test

**Conclusion CLAxon**

Intake of 20 grams of CLA per day revealed no relevant effects on liver- or kidney function in healthy subjects.

## Design of CLARINeT

The design will be equal to that of our earlier studies, e.g. [Mensink, 1990], [Zock, 1995]. It will be a double-blind randomized multiple cross-over trial with 3 treatments:

• CLA

• industrial trans fatty acids (as a positive control)

• oleic acid (Cis 18:1, the monounsaturated fatty acid in unhydrogenated vegetable oils) as a reference fat.

Each volunteer receives each diet for three weeks, in random order, for a total of 9 weeks. Three weeks is sufficient to reach new stable lipoprotein levels.

*Location*: Wageningen University, Division of Human Nutrition, the Netherlands.

Almost all food (90% of energy) is provided to the participants, as described earlier [Mensink, 1990]. Duplicate blood samples are taken in the last week of each dietary period.

The margarines will be produced and coded by NIZO. Analyses of the margarines before the trial will be blinded. The codes will be broken only after the data have been analysed and all authors have agreed on the essential tables and figures.

## Subjects

Sixty generally healthy volunteers between 18 and 65 years of age will be recruited for the study by advertisements and Emails in the Wageningen area. Subjects with chronic disease, such as diabetes or cardiovascular disease, and pregnant and lactating women will be excluded. Other exclusion criteria are total cholesterol (>=6,5 mmol/l) or triglyceride concentrations (>2.3 mmol/l), use of cholesterol lowering medication, use of anti-hypertensive medication, unusual dietary requirements, including high alcohol intakes and BMI > 30. The medical questionnaire at screening will be judged by the health and safety person at the Division of Human Nutrition and if necessary by a medical doctor.

Weights will be measured twice per week and the level of energy intake will be adjusted to compensate for any weight change.

Each subject has to give their written informed consent before screening. Besides, a general, a food frequency and a medical questionnaire have to be filled out.

## Hypothesis, statistical power, and number of subjects

The hypothesis to be tested is:

“There is no difference between the effects of CLA and oleic acid on LDL-cholesterol levels”.

The effect of CLA on LDL will be considered medically relevant if it equals at least half of the effect of industrial trans fatty acids, and CLA may be considered harmless if its effect is less than half of that of industrial trans fatty acids. Such an effect can be picked up at a power of 80% and P=0.05 if 60 subjects are fed diets containing 7% of energy as ALA, oleic or industrial trans fatty acids (about 18g/d)(expected ΛΛ LDL 0.13-0.17 mM versus control; SD ΛΛ LDL 0.35-0.43 mM, n=50-58; appendix 1). The effect of industrial trans fatty acids on LDL and HDL is linear with dose, which suggests that one may extrapolate effects seen at this high dose to the lower intakes in populations (Fig. 2).

Figure 2. The effect of trans fatty acids on the LDL/HDL ratio as a function of the percentage of energy from trans fatty acids in various trials.


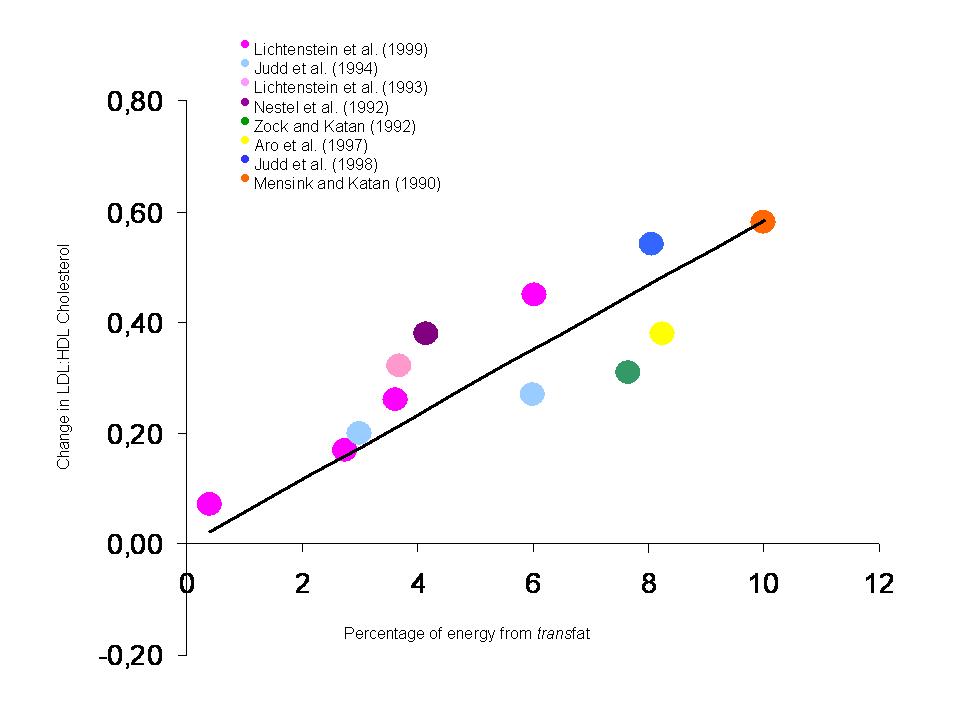


The linear relationship makes clear that a smaller effect could have been picked up if more subjects would be included in the trial. However, for practical reasons we are bound to a maximum of 60 subjects. The 7 energy percent that we provide is in the linear range.

## Diet composition

| Nutrient (% of energy) | Oleic diet | Industrial trans diet | CLA  diet |
| --- | --- | --- | --- |
| Protein | 14 | 14 | 14 |
| Carbohydrates | 45 | 45 | 45 |
| Fat | 40 | 40 | 40 |
| C18:2trans11/cis-9 (t11c9CLA) | 0 | 0 | 7 |
| C18:1trans9/n-9 and other isomers (industrial trans) | 0 | 7 | 0 |
| C18:1cis9/n-9 (Oleic acid) | 23 | 16 | 16 |

The three experimental fats will be incorporated into desserts, yoghurt drinks and margarines, which will also be used for preparation of bread, cookies, sauces and gravies. Other components of the three diets will be identical. Complete daily diets will be provided to subjects; we have a thorough experience in providing diets that meet the experimental requirements and at the same time are tasty and attractive so as to assure compliance. The two experimental trans-containing fats will have about 40% total trans and 60% other fatty acids to avoid problems with digestion. The industrial trans fat will be Fuji FOE 2005-056 (see last page for composition). It has about 40% total trans and is low in dairy trans fatty acids. The CLA fat will be made with the same amount of t11c9CLA as the trans content of the Fuji fat, and the same content of other fatty acids insofar as possible. The position of fatty acids on the triglyceride molecule does not affect their effect on lipoproteins [Zock, 1995]. In the oleic-rich control fat, trans will be replaced by cis. Remaining inequalities between the margarines can be balanced out by adding other fats to the hot meals.

## Analyses

1. Foods and diets:

• Before the trial:

Before the trial fatty acid composition including positional trans isomers will be assessed in the margarines.

During the trial duplicates of each daily diet will be collected, homogenized, stored and analyzed for energy, macronutrients and fatty acid composition.

2. Blood:

- Total and HDL cholesterol, Apo B, LDL cholesterol according to Friedewald
- Triglycerides
- Insulin status markers, such as HOMA and revised QUICKY
- Inflammatory markers, such as C-RP, IL-6, E-selectin, MCP-1, s-TNF-R1, s-TNF-R2, IFg
- Fatty acid composition of cholesteryl esters [Zock, 1997] and erythrocytes as an objective measure of compliance with diets
- Proteomics (In collaboration with Baukje de Roos)

3. Urine: iso-prostanes (in collaboration with Bengt Vessby)

4. Blood Pressure and heart rate

The samples will be stored – coded - for five years to enable us to later repeat analyses or to perform other analyses on indicators of cardiovascular disease, inflammation or insulin status.

## Design of the cross-over study

3 weken

3 weken

3 weken

**cla**

**Oleic acid**

**trans**

measurements


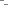


3 weken

3 weken

3 weken

**vacceenr**

measurements


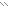


3 weken

3 weken

3 weken

**vacceen**

measurements

**cla**

**cla**

**Oleic acid**

**Oleic acid**

**A**

**B**

**C**

**trans**

**trans**

3 weken

## Statistical analyses

We will only include data of subjects who completed the study and for whom we have complete data (per outcome parameter). Blood collection will take place on two separate days at the end of each diet period. All blood samples of each subject will be analyzed within one run. We average the duplicate measurements in each dietary period and then calculate for each subject the difference between the trans diet and the oleic acid diet and between the CLA diet and the oleic acid diet, so comparisons of the positive control and the CLA against the control treatment (oleic acid). We will test the differences by Mixed Linear Models controlling for order effects. We give two-sided 95% confidence intervals for the differences.

## Investigators

| Name | Function |
| --- | --- |
| Prof Martijn B. Katan PhD | Principal investigator |
| Dr Ingeborg Brouwer | Project leader |
| Dr Baukje de Roos | Co-investigator |
| Els Siebelink RD | Chief dietician |
| Ir Anne Wanders | Junior investigator |
| NN | Junior dieticians |

## Timelines

**May –August 2007:** Preparation of the trial and production of the margarines

**August-September 2007:** Recruitment and selection of the volunteers

**Last week September – Last week November 2007:** Intervention period

**December 2007-March 2008:** Analyses of the blood samples and duplicate diets

**April – Augustus 2008**: Data analyses and writing of manuscript

## Collaboration with Lipid Nutrition

*Timelines*
Lipid Nutrition (Wormerveer, The Netherlands) will deliver the oil in June 2006

*Patents*
No patents are expected, if this study produces patents, they will go to Lipid Nutrition.

*Publication*
All the findings will be published without delay whatever their outcome; at most there could be a delay of 3 months between the time Lipid Nutrition receives the first report and the moment the data may become public.

## References

Ascherio A et al. Trans Fatty Acids and Coronary Heart Disease. New Engl J Med 1999;340:1994.

Baer DJ et al. Dietary fatty acids affect plasma markers of inflammation in healthy men fed controlled diets: a randomized crossover study. Am J Clin Nutr. 2004;79:969-73.

Belury MA, Mahon A, Banni S. The conjugated linoleic acid (CLA) isomer, t10c12-CLA, is inversely associated with changes in body weight and serum leptin in subjects with type 2 diabetes mellitus. J Nutr 2003;133:257S-260S.

Blankson H, Stakkestad JA, Fagertun H, Thom E, Wadstein J, Gudmundsen O. Conjugated linoleic acid reduces body fat mass in overweight and obese humans. J Nutr 2000;130:2943-8.

Gaullier JM, Halse J, Hoye K, et al. Conjugated linoleic acid supplementation for 1 y reduces body fat mass in healthy overweight humans. Am J Clin Nutr 2004;79:1118-25.

Gaullier JM, Halse J, Hoye K, et al. Supplementation with conjugated linoleic acid for 24 months is well tolerated by and reduces body fat mass in healthy, overweight humans. J Nutr 2005;135:778-84.

Ha YL, Grimm NK, Pariza MW. Anticarcinogens from fried ground beef: heat-altered derivatives of linoleic acid. Carcinogenesis 1987;8:1881-7.

Houseknecht KL, Vanden Heuvel JP, Moya-Camarena SY, et al. Dietary conjugated linoleic acid normalizes impaired glucose tolerance in the Zucker diabetic fatty fa/fa rat. Biochem Biophys Res Commun 1998;244:678-82.

Hulshof KF et al. Intake of trans fatty acids in western Europe with emphasis on trans fatty acids: the TRANSFAIR study. Eur J Clin Nutr 1999;53:143-157.

Kamphuis MM, Lejeune MP, Saris WH, Westerterp-Plantenga MS. The effect of conjugated linoleic acid supplementation after weight loss on body weight regain, body composition, and resting metabolic rate in overweight subjects. Int J Obes Relat Metab Disord 2003;27:840-7.

Kuhnt K, Kraft J, Moeckel P, Jahreis G. Trans-11-18:1 is effectively Delta9-desaturated compared with trans-12-18:1 in humans. Br J Nutr 2006; 95: 752-761.

Kreider RB, Ferreira MP, Greenwood M, Wilson M, Almada AL. Effects of conjugated linoleic acid supplementation during resistance training on body composition, bone density, strength, and selected hematological markers. J. Strength Cond. Res. 2002;16:325-34.

Lee KN, Kritchevsky D, Pariza MW. Conjugated linoleic acid and atherosclerosis in rabbits. Atherosclerosis 1994;108:19-25.

Mozaffarian D et al. Trans-Fatty Acids and Cardiovascular Disease. N Engl J Med 2006, 354:1601-13.

Meijer GW et al. Effect of dietary elaidic versus vaccenic acid on blood and liver lipids in the hamster. Atherosclerosis 2001;157:31-40.

Mensink RP, Katan MB. Effect of dietary trans fatty acids on high-density and low-density lipoprotein cholesterol levels in healthy subjects. N Engl J Med. 1990;323:439-45

Park Y, Albright KJ, Liu W, Storkson JM, Cook ME, Pariza MW. Effect of conjugated linoleic acid on body composition in mice. Lipids 1997;32:853-8.

Riserus U, Berglund L, Vessby B. Conjugated linoleic acid (CLA) reduced abdominal adipose tissue in obese middle-aged men with signs of the metabolic syndrome: a randomised controlled trial. Int J Obes Relat Metab Disord 2001;25:1129-35.

Riserus U, Arner P, Brismar K, Vessby B. Treatment with dietary trans10cis12 conjugated linoleic acid causes isomer-specific insulin resistance in obese men with the metabolic syndrome. Diabetes Care 2002;25:1516-21.

Salas-Salvadó J, Marquez-Sandoval F, Bulló M. Conjugated linoleic acid inatke in humans : A systemiatic review focusing on its effect on body composition, glucose, and lipid metabolism. Critical Reviews in Food Science and Nutrition 2006;46:479-88.

Smedman A, Vessby B. Conjugated linoleic acid supplementation in humans--metabolic effects. Lipids 2001;36:773-81.

Taylor JS, Williams SR, Rhys R, James P, Frenneaux MP. Conjugated linoleic acid impairs endothelial function. Arterioscler Thromb Vasc Biol 2006;26:307-12.

Thom E, Wadstein J, Gudmundsen O. Conjugated linoleic acid reduces body fat in healthy exercising humans. J Int Med Res 2001;29:392-6.

Weggemans RM et al. Intake of ruminant versus industrial trans fatty acids and risk of coronary heart disease - what is the evidence? Eur J Lipid Sci Technol 2004;106:390–397.

West DB, Delany JP, Camet PM, Blohm F, Truett AA, Scimeca J. Effects of conjugated linoleic acid on body fat and energy metabolism in the mouse. Am J Physiol 1998;275:R667-72.

Willett WC, et al. Intake of trans fatty acids and risk of coronary heart disease among women. Lancet 1993;341:581-5.

Zock PL et al: Positional distribution of fatty acids in dietary triglycerides: effects on fasting blood lipoprotein concentrations in humans. Am J Clin Nutr 1995;61:48

Zock PL et al. Fatty acids in serum cholesteryl esters as quantitative biomarkers of dietary intake in humans. Am J Epidemiol 1997;145:1114-22.

| Appendix 1: Power table |
| --- |
|  |
| | **Number of subjects required for a comparison of industrial trans with CLA, power 80%, P=0.05, n=7.9 x (SD/D)^2** | | | | | | | | |  | | --- | --- | --- | --- | --- | --- | --- | --- | --- | --- | |  |  |  |  |  |  |  |  |  |  | | **LDL** |  |  |  |  |  |  |  |  |  | | SD /\ LDL (Mensink & Katan 1990) | 0,35 |  |  |  |  |  |  |  |  | | en% industrial trans | 10 | 9 | 8 | 7 | 6 | 5 | 4 | **3,5** | 2 | | Effect on LDL (mmol/L) | 0,37 | 0,33 | 0,30 | 0,26 | 0,22 | 0,19 | 0,15 | **0,13** | 0,07 | | Number of subjects needed | 7 | 9 | 11 | 14 | 20 | 28 | 44 | **58** | 177 | |  |  |  |  |  |  |  |  |  |  | | SD /\ LDL (Mensink & Katan 1990) | 0,35 |  |  |  |  |  |  |  |  | | en% vaccenic acid | 10 | 9 | 8 | 7 | 6 | 5 | 4 | **3,5** | 2 | | Effect (mmol/L) if effect vaccenic is half of effect industrial trans | 0,19 | 0,17 | 0,15 | 0,13 | 0,11 | 0,09 | 0,07 | **0,06** | 0,04 | | Number needed if effect vaccenic is half of effect industrial trans | 28 | 35 | 44 | 58 | 79 | 113 | 177 | **231** | 707 | |  |  |  |  |  |  |  |  |  |  | | SD LDL (Mensink 2005, pers comm) | 0,43 |  |  |  |  |  |  |  |  | | en% industrial trans | 10 | 9 | 8 | 7 | 6 | 5 | 4 | **3,5** | 2 | | Effect on LDL, meta-anal 2003 | 0,49 | 0,44 | 0,39 | 0,34 | 0,29 | 0,25 | 0,20 | **0,17** | 0,10 | | Number of subjects needed | 6 | 8 | 10 | 12 | 17 | 24 | 38 | **50** | 152 | |  |  |  |  |  |  |  |  |  |  | | SD LDL (Zock et al 1992) | 0,45 |  |  |  |  |  |  |  |  | | en% industrial trans | 10 | 9 | 8 | 7 | 6 | 5 | 4 | **3,5** | 2 | | Effect on LDL (mmol/L) | 0,37 | 0,33 | 0,30 | 0,26 | 0,22 | 0,19 | 0,15 | **0,13** | 0,07 | | Number of subjects needed | 12 | 14 | 18 | 24 | 32 | 47 | 73 | **95** | 292 | |  |  |  |  |  |  |  |  |  |  | | **HDL** |  |  |  |  |  |  |  |  |  | | SD /\ HDL (Mensink & Katan 1990) | 0,16 |  |  |  |  |  |  |  |  | | en% industrial trans | 10 | 9 | 8 | 7 | 6 | 5 | 4 | **3,5** | 2 | | D (mmol/L) | -0,17 | -0,15 | -0,14 | -0,12 | -0,10 | -0,09 | -0,07 | **-0,06** | -0,03 | | n | 7 | 9 | 11 | 14 | 19 | 28 | 44 | **57** | 175 | |  |  |  |  |  |  |  |  |  |  | | SD /\ HDL (Mensink 2005, pers comm) | 0,14 |  |  |  |  |  |  |  |  | | en% industrial trans | 10 | 9 | 8 | 7 | 6 | 5 | 4 | **3,5** | 2 | | Effect on HDL, meta-anal 2003 | -0,08 | -0,07 | -0,06 | -0,06 | -0,05 | -0,04 | -0,03 | -0,03 | -0,02 | | n | 32 | 39 | 49 | 64 | 88 | 126 | 198 | **258** | 790 | |  |  |  |  |  |  |  |  |  |  | | SD /\ HDL (Zock et al 1992) | 0,16 |  |  |  |  |  |  |  |  | | en% industrial trans | 10 | 9 | 8 | 7 | 6 | 5 | 4 | **3,5** | 2 | | D (mmol/L) | -0,17 | -0,15 | -0,14 | -0,12 | -0,10 | -0,09 | -0,07 | **-0,06** | -0,03 | | n | 7 | 9 | 11 | 14 | 19 | 28 | 44 | **57** | 175 | |  |  |  |  |  |  |  |  |  |  | | **95% CI:** | M&K ΔLDL | M&K ΔHDL | Zock92 ΔLDL | Zock92 ΔHDL | Zock92 ΔHDL |  |  |  |  | | lowest | 0,28 | 0,13 | 0,12 | 0,03 | 0,06 |  |  |  |  | | highest | 0,45 | 0,2 | 0,35 | 0,1 | 0,13 |  |  |  |  | | SE | 0,04 | 0,02 | 0,06 | 0,02 | 0,02 |  |  |  |  | | n | 59 | 59 | 56 | 56 | 56 |  |  |  |  | | SD | 0,33 | 0,14 | 0,44 | 0,13 | 0,13 |  |  |  |  | |

##

## Appendix 2: available partially hydrogenated industrial fats

| **Samples** | **Fuji** | **Fuji** | **Fuji** | **Fuji** | **Fuji** |
| --- | --- | --- | --- | --- | --- |
|  | **Mélano H 350 G (octobre 2004)** | **FOE 2005-052** | **FOE 2005-053** | FOE 2005-056 | **FOE 2005-057** |
| FA profile (%) |  |  |  |  |  |
|  |  |  |  |  |  |
| C12:0 | 0.3 | 0.8 | 0.9 | 0.7 | 0.6 |
| C14:0 | 0.5 | 1.2 | 0.8 | 1.2 | 0.8 |
| C16:0 | 17.4 | 38.0 | 22.1 | 35.2 | 24.1 |
| C18:0 | 6.1 | 5.1 | 7.0 | 4.7 | 6.1 |
| C18:1c9 | 3.9 | 21.8 | 27.6 | 14.6 | 16.5 |
| C18:1c11 | 2.1 | 1.0 | 1.5 | 1.1 | 1.7 |
| C18:1t4 | 0.4 | 0.0 | 0.1 | 0.0 | 0.3 |
| C18:1t5 | 1.1 | 0.1 | 0.4 | 0.1 | 0.9 |
| C18:1t6+t7+t8 | 3.8 | 5.2 | 7.9 | 7.3 | 11.5 |
| C18:1t9 | 6.9 | 10.6 | 6.1 | 15.4 | 7.0 |
| C18:1t10 | 10.1 | 5.6 | 6.0 | 7.6 | 6.7 |
| C18:1t11 | 7.4 | 2.6 | 3.4 | 3.3 | 5.6 |
| C18:1t12 | 11.8 | 0.0 | 4.2 | 2.1 | 7.2 |
| C18:1t13t14 | 16.8 | 2.4 | 4.6 | 3.4 | 8.7 |
| C18:1t15 | 1.5 | 2.0 | 2.1 | 2.8 | 2.9 |
| C18:1t16 | 1.0 | 0.1 | 0.9 | 0.1 | 1.5 |
| C18:2 | 0.1 | 1.1 | 4.6 | 0.7 | 1.4 |
| C18:2:t10 | 0.0 | 0.0 | 0.0 | 0.0 | 0.0 |
| C18:2t11 | 0.0 | 0.0 | 0.0 | 0.0 | 0.0 |
| C18:3 | 0.0 | 0.1 | 0.3 | 0.1 | 0.1 |

|  |  |  |  |  |  |
| --- | --- | --- | --- | --- | --- |
| **Sums** |  |  |  |  |  |
| Saturated fatty acids | 24.3 | 45.0 | 30.8 | 41.9 | 31.6 |
| cis-monounsaturated fatty acids | 6.0 | 22.8 | 29.1 | 15.8 | 18.2 |
| C18:1 trans | 60.7 | 28.5 | 35.6 | 42.0 | 52.3 |
| polyunsaturated fatty acids | 0.1 | 1.2 | 4.9 | 0.7 | 1.5 |
| C18:2 trans | 0.0 | 0.0 | 0.0 | 0.0 | 0.0 |
| (Checksum) | 91.2 | 97.6 | 100.4 | 100.3 | 103.6 |
|  |  |  |  |  |  |
| **C18:1 TFA profile (% of total C18:1 TFAs)** |  |  |  |  |  |
|  |  |  |  |  |  |
| C18:1t4 | 0.7 | 0.0 | 0.4 | 0.0 | 0.6 |
| C18:1t5 | 1.7 | 0.2 | 1.0 | 0.2 | 1.7 |
| C18:1t6+t7+t8 | 6.3 | 18.4 | 22.2 | 17.3 | 21.9 |
| C18:1t9 (elaidic acid) | 11.3 | 37.3 | 17.2 | 36.7 | 13.5 |
| C18:1t10 | 16.7 | 19.6 | 16.7 | 18.0 | 12.8 |
| C18:1t11(vaccenic acid) | 12.2 | 9.0 | 9.7 | 7.9 | 10.7 |
| C18:1t12 | 19.5 | 0.0 | 11.7 | 4.9 | 13.7 |
| C18:1t13t14 | 27.6 | 8.3 | 12.9 | 8.1 | 16.7 |
| C18:1t15 | 2.4 | 6.9 | 5.8 | 6.7 | 5.6 |
| C18:1t16 | 1.7 | 0.2 | 2.5 | 0.2 | 2.9 |
| (checksum) | 100.0 | 100.0 | 100.0 | 100.0 | 100.0 |
